# Advanced Ultrasound Screening for Temporomandibular Joint (TMJ) Internal Derangement

**DOI:** 10.1155/2020/1809690

**Published:** 2020-05-04

**Authors:** Saul N. Friedman, Miriam Grushka, Hussam K. Beituni, Madhu Rehman, Hart B. Bressler, Lawrence Friedman

**Affiliations:** ^1^Division of Nuclear Medicine, Mallinckrodt Institute of Radiology, Washington University School of Medicine, Saint Louis, MO 63110, USA; ^2^974 Eglington Avenue West, Toronto, Ontario M6C 2C5, Canada; ^3^Department of Medical Imaging, North York General, 4001 Leslie Street, Toronto, Ontario M2K 1E1, Canada; ^4^Department of Family and Community Medicine, University of Toronto, Mount Sinai Hospital, 600 University Ave, Toronto, Ontario M5G 1X5, Canada

## Abstract

**Purpose:**

To present an advanced ultrasound (US) technique and propose its use as a screening diagnostic tool for temporomandibular joint (TMJ) internal derangement.

**Materials and Methods:**

The technique is based on maintaining the US probe parallel to the articular disc, rather than traditional axial and coronal views, with the position described relative to a clock face. Validation was achieved by direct comparison with magnetic resonance imaging (MRI). A total of 61 patients, with age ranging from 13 to 67 years, were prescreened for TMJ pain and internal derangement, underwent US imaging for screening, and MRI evaluation for final diagnosis.

**Results:**

29 of the 61 patients had disc pathology on MRI. US screening produced no false positive results and only 6 false negative results, corresponding to a sensitivity of 79% and specificity of 100%. Half of the false negative cases involved disc pathology with a medial component to the disc displacement.

**Conclusion:**

US is both a sensitive and a specific screening tool for TMJ dysfunction when used by an appropriately trained operator, with the exception of medially displaced discs. If TMJ assessment is found to be abnormal, the patient should be referred for MRI, and any patient scheduled for surgery must have the diagnosis confirmed by MRI. If a component of medial disc displacement is suspected, MRI should be performed despite a normal screening US.

## 1. Introduction

In the past, internal derangement of the temporomandibular joint (TMJ) has been assessed with plain film radiographs as the initial investigation [1] followed by arthrography [2] and computed tomography (CT) [3]. More recently, magnetic resonance imaging (MRI) [4–7] has provided a noninvasive accurate method of assessing the TMJ without associated radiation risks. Its advantages over previous methods include its ability to directly visualize the disc and accurately determine the position of the disc with respect to the condyle of the mandible and eminence of the temporal bone. The examination, however, takes 20 to 45 minutes on average to perform depending on the scanner and protocol, and patients have difficulty keeping still and having their mouths open for extended periods of time, especially if they are experiencing pain. In addition, the examination is costly and access is still limited in many centres. Many patients also experience claustrophobia and are unable to complete or even undergo the examination.

Ultrasound is relatively inexpensive, is readily accessible, and can be performed in most outpatient facilities; studies take only an average of 10 to 15 minutes in total, and it is without any known risks. In addition, US provides the opportunity to converse with patients and identify the exact locations of pain, while the probe can be used as a palpable tool for real-time identification of crepitus, clicking, motion, and snapping sensations. The disadvantages of US remain the long learning curve and the fact that the test is operator dependent. There is also the question of ultrasound being effectively used as a diagnostic tool [8–13], given the many constraints that will be discussed. In particular, Katzberg [12] questioned its validity stating that ultrasound images do not appear to be anatomically correct, that tissues indicated to be discs were not convincing, and that interpretation was not blinded. He also believed that given the bone blockade barrier and inability of the ultrasound beam to bend around curved narrow structures, ultrasound does not allow for adequate diagnosis. However, more recently, Katzberg and colleagues have proposed the use of sagittal transoral US to evaluate the TMJ [14].

Additional and more recent works by numerous investigators provide strong support for the use of conventional US techniques. For example, Li et al. [13] in 2012 calculated a sensitivity and specificity of 76% and 82% for disc displacement with reduction, respectively, and 79% and 91% without disc reduction, respectively. They also stated that ultrasound is acceptable as a rapid initial method to exclude some potential patients but recommended MRI if treatment is necessary. In addition, they noted that the ability to diagnose lateral and posterior displacements by ultrasound is not clear. More recently, Su et al. published a meta-analysis in 2018 noting an overall sensitivity and specificity of 75–81% and 73–80%, respectively, for disc displacement in the closed-mouth position and 65–74% and 86–91%, respectively, for disc displacement in the open-mouth position [15].

Katzberg [12] attempted to quantify the distribution of disc displacements in a prospective study of 76 volunteers and 102 patients studied with MRI. He noted 52% of abnormal joints had anterior displacement, 27% of abnormal joints had anteromedial displacement, 12% of abnormal joints had medial displacement, 34% of abnormal joints had anterolateral displacement, and 4% of abnormal joints had lateral displacement. He also stated that anteromedially and medially displaced discs, totalling 39%, would not be visualized by ultrasound.

### 1.1. Goal

Using knowledge of the normal US appearance and dynamic motion of the articular disc, we present a new technique for visualization and assessment of the TMJ using ultrasound, where the probe is positioned parallel to the disc throughout movement; this technique differs from traditional vertical and horizontal probe positioning, where the focus has been on obtaining true axial and coronal plains of imaging. MRI of the TMJ is used as the gold standard for technique validation.

## 2. Materials and Methods

Institutional review board approval from North York General Ethics Committee was obtained, and verbal consent was obtained from all patients and subjects prior to inclusion in the study.

### 2.1. Ultrasound Technique Details

The patient can be in a sitting position or lying down on a stretcher. We prefer the patients lying supine with the jaw tilted away from the side to be examined, as this position not only offers more stability for probe positioning but also allows for further patient comfort. The joint is then palpated while we have the patient open and close their mouth. Once the joint is located, the probe is placed over the joint using a liberal amount of warm gel.

**Figure 1:**
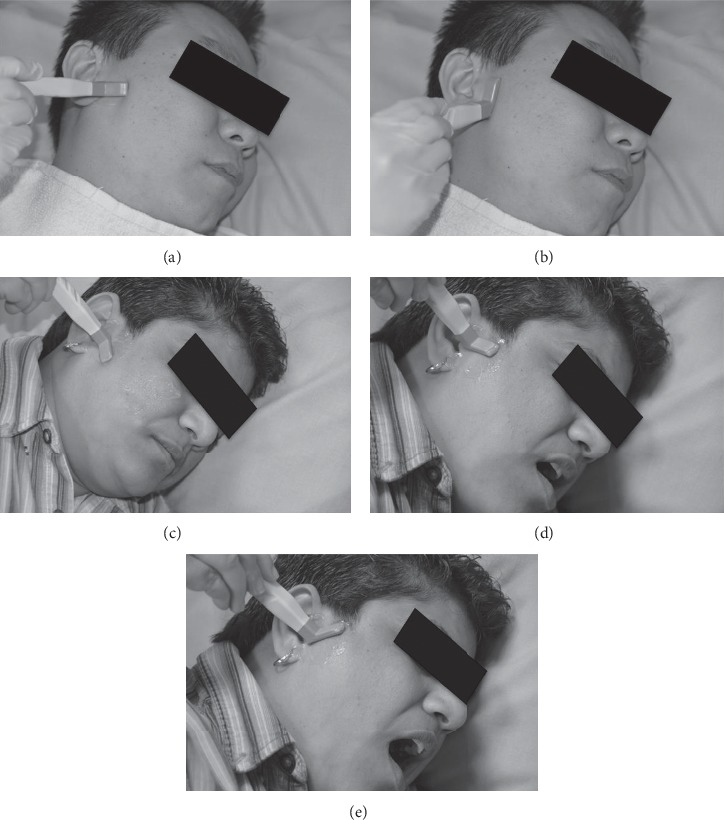
Position of the probe on the skin surface for traditional (a) transverse and (b) coronal scans. The probe position during routine interrogation of the TMJ is (c) angled parallel to the disc in the sagittal plane at 50–60° below the horizontal as measured from the anterior side of the probe or 5 o'clock position in the closed-mouth position on the right, (d) angled parallel to the disc or 3 o'clock position in the half-open-mouth position on the right, and (e) angled 50–60° above the horizontal as measured from the anterior side of the probe in the fully open-mouth or 1 o'clock position on the right. In a similar manner, these angles represent 7 o'clock, 9 o'clock, and 11 o'clock positions when investigating the TMJ on the left.

Traditional ultrasound techniques have included positioning of the probe on the skin surface in the axial (Figure 1(a)) and coronal (Figure 1(b)) planes [12, 13]. We employ a method whereby the position of the probe can be compared to that of a clock face in a clockwise rotation. The probe is angled appropriately parallel to the right disc in the sagittal plane initially at an angle 50–60° down from the horizontal as measured from the anterior side of the probe or 5 o'clock in the closed-mouth position on the right (Figure 1(c)), followed by 0° angulation or 3 o'clock in the semiopen position (Figure 1(d)), and finally an anterior angle of 50–60° up from the horizontal as measured from the anterior side of the probe or 1 o'clock in the fully open-mouth position on the right (Figure 1(e)). In a similar manner, these angles would correspond to 7 o'clock, 9 o'clock, and 11 o'clock positions, respectively, when investigating the left TMJ. Dynamic video clips of movement can be obtained by swivelling the probe or by keeping the probe just off the horizontal, which varies from patient to patient.

The disc was considered normal in the static closed-mouth position if the intermediate zone or centre of the hypoechoic disc was located between the anterosuperior aspect of the condyle and the posteroinferior aspect of the articular eminence. During movement, the disc was considered normal if the intermediate zone or centre of the disc maintained its central location to the condyle from the closed-mouth to fully open-mouth positions. All other disc locations were considered to exhibit either anterior or posterior displacement.

### 2.2. Imaging Equipment

Ultrasound images were obtained using a 15–7 MHz L15-7io hockey stick transducer (Philips iU22 Ultrasound System, Netherlands). The total US image acquisition time was approximately 10 minutes per patient.

MRI was performed using T1 coronal and sagittal images in both open- and closed-mouth positions as well as gradient kinetic dynamic sagittal images (GE Signa HDxt 1.5T, Milwaukee, USA) with the following parameters: coronal T1-weighted images with repetition time (TR) = 405 ms and echo time (TE) = 10 ms, sagittal proton-density-weighted images with TR = 2025 ms and TE = 30 ms, and sagittal T2^∗^-weighted kinematic dynamic images with TR = 100 ms and TE = 10 ms. The total acquisition time was approximately 30 minutes per patient.

### 2.3. Validation with Normal Volunteers

Normal anatomy was first reviewed through the evaluation of both TMJs in 10 normal volunteers between the ages of 20 and 30 years.

### 2.4. Assessment of Patient Pathology

Patients were prescreened for TMJ pain and internal derangement based on the history and clinical exam by our TMJ pathology specialist (author MG) or our pain specialist with particular experience in TMJ pathology (author HBB). Recommendations on the physical exam technique have been described in detail elsewhere [16]. A total of 61 symptomatic patients were recruited, of which 43 were male and 18 female with a mean age of 40 years and age range of 13 to 67 years. MR and US imaging was obtained using the same equipment and protocol as above. All US images were acquired by the senior radiologist (author LF), who had over 20 years of specialized US MSK experience. Contrast was not used for either MR or US imaging. MRI interpretations were performed by 4 radiologists, each with a minimum of 5 years of experience working in a practice, and typically completed first to avoid delay in patient care. The same senior MSK radiologist with over 20 years of specialized US MSK training performed all US interpretations. To avoid recall bias, the US reader remained blinded to all MRI results performed by other readers. In addition, in the situation where the US reader also interpreted the MRI first, a period of 3-4 weeks was waited after the MRI interpretation before interpreting the associated US.

The US screen was considered positive if displacement was observed, regardless of the direction (anterior, posterior, medial, or lateral) and regardless of whether it reduced or remained fixed. In cases where displacement was observed in multiple directions (e.g., anteromedial), the displacement was labelled by the predominant direction of displacement. Pathology was then further subdivided into fixed displacement and recapture subgroups. Osteoarthritic changes and evidence of inflammation as determined through increased blood flow were also evaluated but could only be performed on US as MRI postcontrast imaging was not performed.

## 3. Results

### 3.1. Normal TMJ

**Figure 2:**
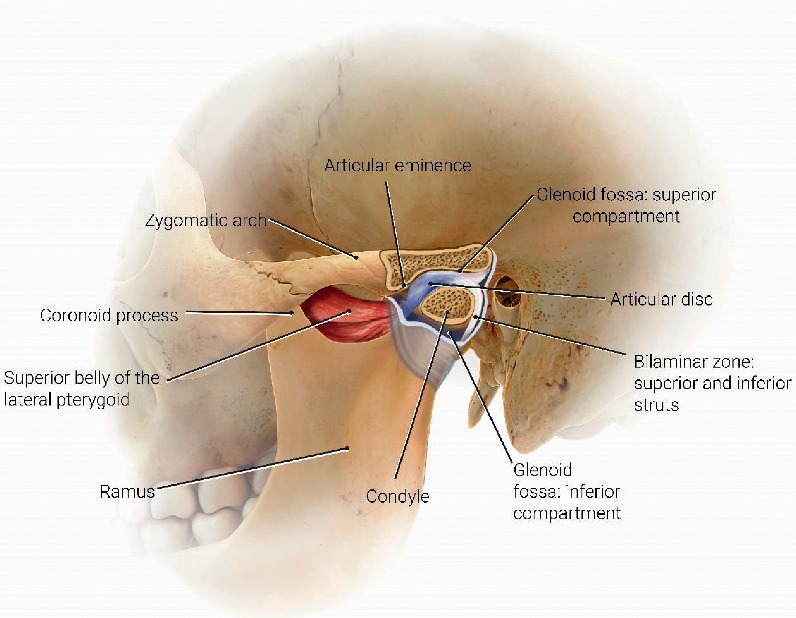
Normal anatomy illustrating the condyle of the mandible, superior and inferior belly of the lateral pterygoid muscle, articular disc, articular eminence of the temporal bone, and articular fossa (glenoid fossa) of the temporal bone.

A diagram of basic anatomy is presented in Figure 2 as a visual aid. The articular disc in the normal TMJ is a fibrous structure composed of a superficial band, a slightly more thickened deep band, and a very thin intermediate zone that is attached posteriorly to the temporal bone through the bilaminar zone and anteriorly to the lateral pterygoid muscle superior belly. A lower synovial-lined joint space separates it from the head of the condyle, and a noncommunicating upper joint separates it from the bony glenoid fossa and the articular eminence of the temporal bone.

**Figure 3:**
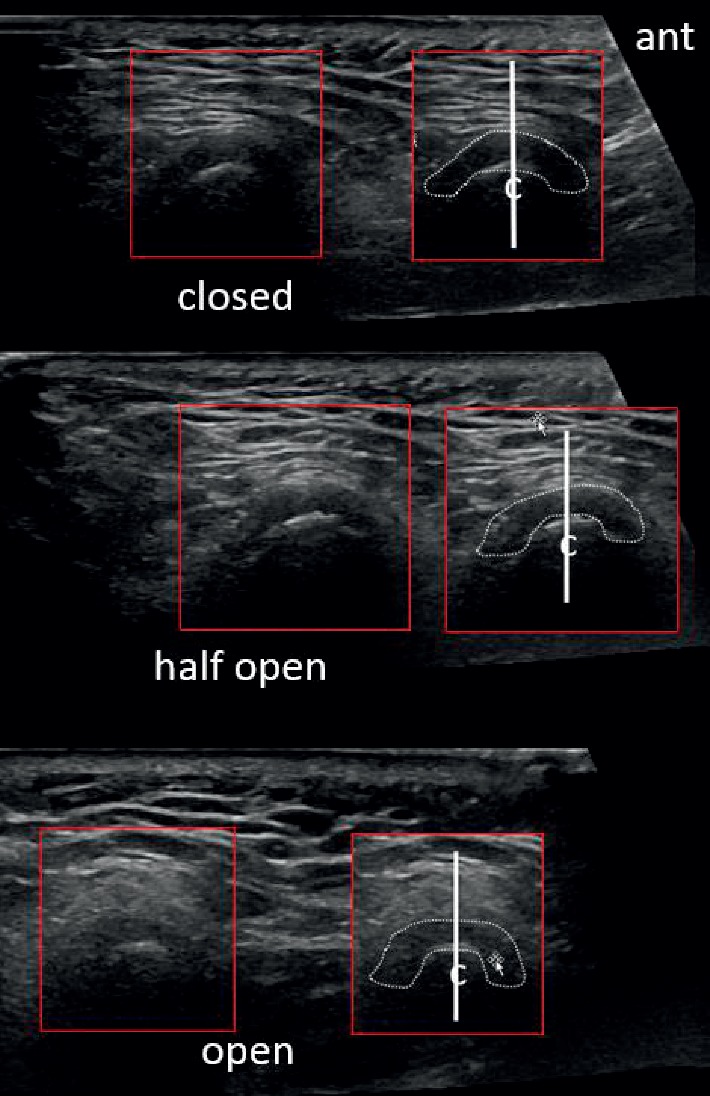
Normal ultrasound appearance of the articular disc in the sagittal plane, seen as an inverted hypoechoic c-shaped structure. Note that the disc maintains a constant central appearance with respect to the centre of the condyle, “c,” outlined by a central vertical line during the closed-mouth, half-open-mouth, and fully-open-mouth views. The anterior (“ant”) band and posterior band of the articular disc appear symmetrical in size with respect to the centre of the condyle. A focal annotated view was provided to aid visualization with the articular disc outlined with a dotted contour.

On MRI, the disc has a bowtie or saddle appearance. On sonography, a normal disc usually appears as an inverted more hypoechoic c-shaped structure that straddles the hyperechoic cortex of the condyle, as seen in Figure 3. However, the disc can also appear isoechoic to hyperechoic especially as it becomes less hydrated and more calcified with disease.

During normal movement and disc translation, the disc maintains a constant relationship between the condyle head of the mandible and the eminence of the temporal bone. From the closed-mouth to the fully-open-mouth position, the thin intermediate zone should be between the closely applied cortical surface of the condyle and the eminence. Close attention to this relationship is critical in separating a normal from an abnormal disc on both MRI and ultrasound interrogation. For a right-sided TMJ in the closed-mouth position in the sagittal plane, the anterior angle of the normal disc is 50–60° down from the horizontal. When midway between open- and closed-mouth positions, the anterior angle is 0° to the horizontal, and in the fully-open-mouth position, it is 50–60° up from the horizontal. The mirror image configuration is present in the left-sided TMJ.

### 3.2. Abnormal TMJ

Pathology for the 61 patients included in the study as determined by MRI is summarized in Table 1. The 29 patients positive for TMJ pathology correspond to a prevalence of 47.5%. Fixed posterior displacement was not observed. Of the 14 patients with fixed anterior displacement, 3 were noted to have a medial component to the displacement. Lateral disc displacement was not observed.

Ultrasound screening identified 23 of the 29 patients with pathology, corresponding to 6 false negatives. No false positive results were obtained. This corresponds to a sensitivity of 79% and specificity of 100%; results are summarized in Table 2. The 6 false negative cases all corresponded to fixed anterior displacement and included all 3 cases that were noted to contain a medial component of displacement. Based on the calculated prevalence of 47.5%, the calculated positive predictive value is 100% and the negative predictive value is 84%.

**Figure 4:**
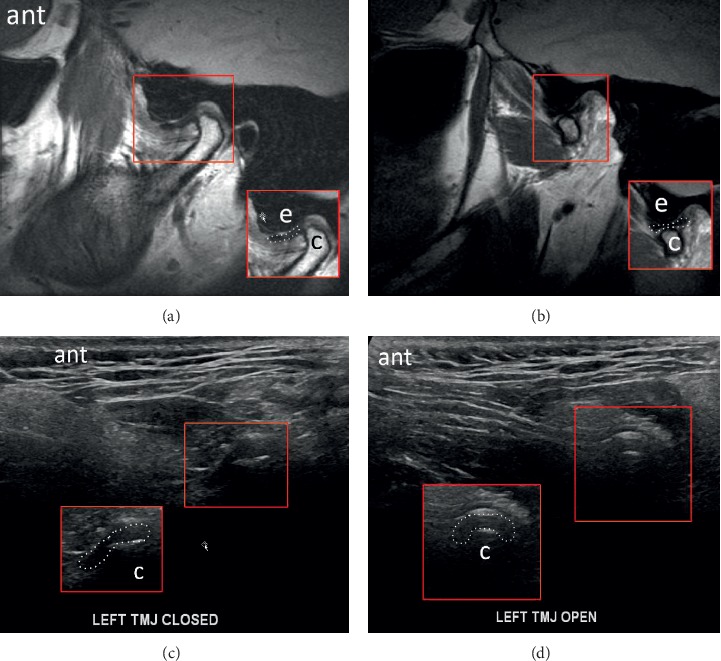
Images demonstrating anterior displacement of the disc in the sagittal plane in the (a) closed-mouth position with MR, (b) disc recapture in the open-mouth position with MR, (c) closed-mouth position with US, and (d) recapture in the open-mouth position with US. The disc retains normal morphology. A focal annotated view was provided to aid visualization with the articular disc outlined with a dotted contour; condyle = “c”; eminence = “e”; anterior = “ant.”

**Figure 5:**
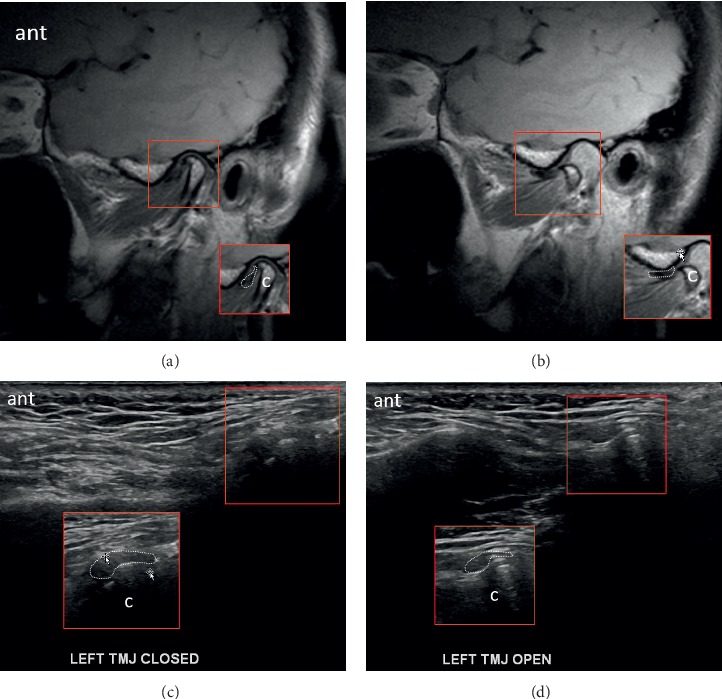
Images demonstrating a deformed disc with fixed anterior dislocation in the sagittal plane in the (a) closed-mouth position with MRI, (b) open-mouth position with MRI, (c) closed-mouth position with US, and (d) open-mouth position with US. A focal annotated view was provided to aid visualization with the articular disc outlined with a dotted contour; condyle = “c”; anterior = “ant”; left = “LT.”

**Figure 6:**
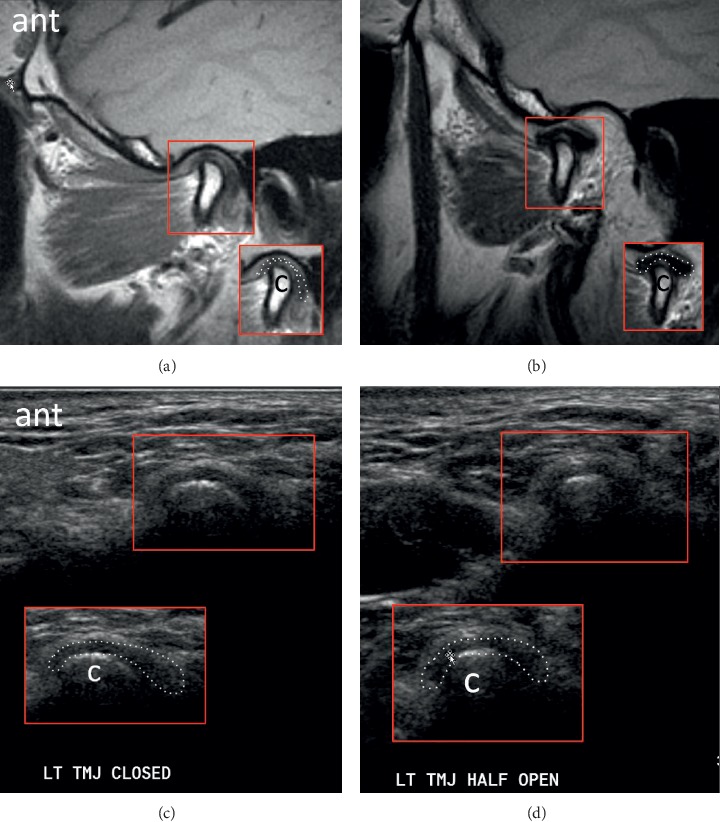
Images demonstrating posterior displacement of the disc in the (a) closed-mouth position with MRI, (b) recapture in the open-mouth position with MRI, (c) closed-mouth position with US, and (d) recapture in the open-mouth position with US. A focal annotated view was provided to aid visualization with the articular disc outlined with a dotted contour; anterior = “ant”; condyle = “c”; left = “LT.”

**Figure 7:**
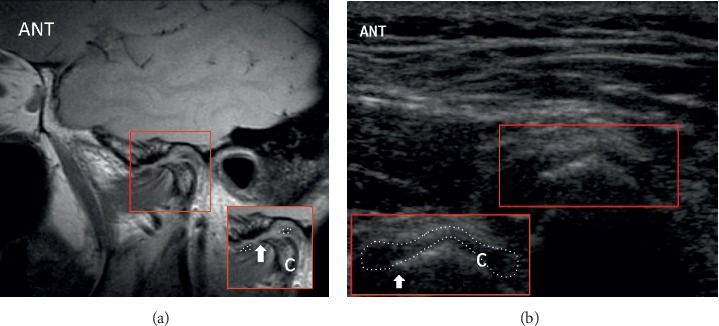
Osteoarthritis with the deformed articular surface of the condyle with a large anterior marginal osteophyte and deformed disc with central thinning on (a) MR and (b) ultrasound. A focal annotated view was provided to aid visualization with the articular disc outlined with a dotted contour and the osteophyte indicated by an arrow; anterior = “ANT”; condyle = “c.”

US and MRI of anterior disc displacement with recapture of the disc in the open-mouth position are demonstrated in Figure 4. Note the normal MRI bowtie appearance in Figure 4(b). Corresponding pathology without recapture, labelled as fixed anterior dislocation, is demonstrated in Figure 5. While the morphology of the disc usually remains normal with an anteriorly displaced disc that recaptures, it more often appears morphologically abnormal with fixed anterior dislocation, as noted in Figure 5. Posterior displacement is demonstrated in Figure 6. Bony deformity and bony osteophyte formation in osteoarthritis involving the condyle can be visualized on ultrasound, as demonstrated in Figure 7.

## 4. Discussion

We introduce an advanced technique for evaluating the temporomandibular joint through ultrasound imaging by positioning the probe parallel to the articular disc throughout dynamic motion. US was presented as a screening tool for TMJ dysfunction due to its ubiquitous nature and relatively inexpensive cost. MRI was chosen as the gold standard for comparison due to its high level of specificity and sensitivity, as well as its role in current standard of practice.

We have limited our description of articular disc pathology to anterior, posterior, medial, and lateral dislocation, based on the most prominent feature only. While this creates labelling bias, this is unlikely to affect the patient outcome as the primary role is screening and subsequent MRI studies were performed for further characterization. Lateral displacement was not visualized in our study; however, this is not unexpected. Based on the 4% prevalence of lateral displacement by Katzberg et al. [17], only a single case is expected within a group of 29, and random chance alone is enough to explain its absence. However, medial and lateral displacements were generally difficult to visualize on US and are likely underestimated based on US alone. Validation of lateral displacement on US will require a much larger sample size given the relative rarity.

Prescreening patients for TMJ symptoms inherently creates a selection bias, with our measured prevalence of 47.5% grossly overestimating prevalence in the general population, but reflecting realistic clinical practice and the likely imaging prevalence. While the power of our study is limited, we believe our results to be sufficient for the initial validation of US as a screening tool to help decide who should proceed with additional MRI. We continue to screen patients with our US technique and have evaluated more than 350 additional patients; however, lack of corresponding MRI evaluation prevented their inclusion in this study.

Ethical management, requiring a definitive diagnosis for treatment to not be delayed, prevented US image acquisition and interpretation prior to MRI evaluation. Potential bias was minimized by limiting the number of MRIs interpreted by the expert radiologist who interpreted the US studies, and keeping him ignorant of MRI results until assessment of the US images was completed. In cases where the MRI was read first by the same radiologist who read the subsequent US, the US interpretation was held for 3-4 weeks to minimize recall bias.

Of the 6 false negative cases obtained using our technique, all cases noted to involve medial disc displacement were included. This is likely due to the limited penetrance of US because of bone and other anatomy, restricting it to the superficial one-third of the disc. Others have also noted that laterally or medially displaced discs are known to be inadequately visualized and evaluated with present ultrasound techniques [13].

Advantages of US over MRI identified during our study include the ability to observe real-time motion, clicking and crepitus, the ability to localize imaging to patient-directed regions of pain, and the ability to evaluate patients who are claustrophobic or have stents and implants that are not MRI compatible. Doppler imaging enabled us to diagnose inflammation by the presence of abnormal blood flow that, while theoretically possible if contrast is used, is unlikely on MRI with standard practices. US is also able to visualize cortical osseous defects including osteophytes and erosions. Retrodiscal visualization with US may partially be limited by patient body habitus and permitted acoustic windows by the adjacent osseous structures.

Disadvantages of US include the previously mentioned difficulties in visualizing medial and lateral disc displacements. While disc thickness and shape can be assessed with US, perforations and adhesions will not be adequately visualized. Subcortical osseous abnormalities also cannot be visualized. US always carries the inherent operator dependence, and there is necessarily a learning curve before an operator will obtain the expertise required to match the results presented in this paper. Our study is also limited to conventional US and MRI reconstruction algorithms. Others have evaluated enhancement filters for possible improvement of diagnostic efficiency on MRI [18], and similar evaluation on US is a possible topic for future research.

Our referring clinicians have stressed the importance of TMJ imaging to help differentiate between early and late changes. Early changes tend to be limited to disc displacement only, while late changes tend to include osseous remodelling, which may be underestimated or not assessed despite the advanced experience of the clinician. Fixed versus reducible disc displacements were important to them for treatment planning. For fixed disc displacement, our clinicians focus on mobilization of the joint, while reducible displacements are usually treated with bite splints. Surgery was not a common treatment option in their practices.

We believe that US ultimately has a role beyond screening for further MRI evaluation and can be used for definitive diagnosis in the majority of cases; however, definitive validation will likely require further evaluation using a larger sample size and hopefully a randomized prospective study in which patients can be placed in MRI-only and US-only evaluation.

## 5. Conclusions

US screening is a viable supplement to the more expensive MRI evaluation, especially when MRI access is limited, with the following caveats:

1. Ultrasound should currently be limited to use as an initial screening tool, and the sonologist needs to only detect if the exam is normal or abnormal.
2. If found to be normal, and surgery is not contemplated, then no further investigation is deemed necessary.
3. If found to be abnormal, the patient should be referred for MRI.
4. Due to poor sensitivity in visualizing medial disc displacement, if suspected, MRI should be performed despite a normal screening US.

## Figures and Tables

**Table 1:** A total of 29 of the 61 patients experiencing temporomandibular symptoms had pathology visible on magnetic resonance imaging (MRI), corresponding to a disease prevalence of 47.5%. Pathology is subdivided into anterior, posterior, and medial displacements and summarized here, with the number demonstrating the presence of recapture, osteoarthritic changes, and presence of inflammation (as determined from ultrasound imaging) noted.

Pathological disc displacement	Fixed	Recapture present	Total
Anterior	14^*∗*^	14	28
Posterior	0	1	1
Medial	0	0	0
Lateral	0	0	0
Total	14	15	29

^*∗*^3 cases were further noted to have a medially displaced component.

**Table 2:** Summary of results of the 61 patients prescreened for temporomandibular symptoms using ultrasound (US) and compared to the gold standard of magnetic resonance imaging (MRI). US results are split into true positive and true negative (concordant with MRI), and false positive and false negative (discordant with MRI), corresponding to a sensitivity of 79% and specificity of 100%.

	MRI screened positive	MRI screened negative
US screened positive	23	0
US screened negative	6	32

## Data Availability

A summary of image interpretation is provided with appropriate data provided in the manuscript. Select images are provided in the manuscript. Each individual case image is not available.
